# Hypoxia Promotes Syndecan-3 Expression in the Tumor Microenvironment

**DOI:** 10.3389/fimmu.2020.586977

**Published:** 2020-09-30

**Authors:** Endika Prieto-Fernández, Leire Egia-Mendikute, Alexandre Bosch, Ana García del Río, Borja Jimenez-Lasheras, Asier Antoñana-Vildosola, So Young Lee, Asis Palazon

**Affiliations:** ^1^Cancer Immunology and Immunotherapy Lab, Centre for Cooperative Research in Biosciences CIC bioGUNE, Basque Research and Technology Alliance, Derio, Spain; ^2^Ikerbasque, Basque Foundation for Science, Bilbao, Spain

**Keywords:** syndecan-3, hypoxia, solid tumors, tumor microenvironment (TME), cancer immunotherapy

## Abstract

The syndecan (Sdc) family is comprised of four members of cell surface molecules (Sdc-1 to 4) with different biological functions. Syndecan-3 (Sdc-3) is known to be mainly expressed in the brain and nervous tissue and plays a key role in development, cell adhesion, and migration. Recent studies point to important roles for Sdc-3 in inflammatory disease, but the patterns of expression and significance of Sdc-3 in cancer remains unexplored. Here we show that Sdc-3 expression is upregulated on several cancer types, especially in solid tumors that are known to be hypoxic. The Cancer Genome Atlas program (TCGA) data demonstrated that Sdc-3 expression in the tumor microenvironment positively correlates with a hypoxia gene signature. To confirm a potential cause-effect, we performed experiments with tumor cell lines showing increased expression upon *in vitro* exposure to 1% oxygen or dimethyloxalylglycine, an inhibitor of prolyl hydroxylases, indicating that Sdc-3 expression is promoted by hypoxia inducible factors (HIFs). HIF-1α was responsible for this upregulation as confirmed by CRISPR-engineered tumor cells. Using single-cell RNA sequencing data of melanoma patients, we show that Sdc-3 is expressed on tumor associated macrophages, cancer cells, and endothelial cells. Syndecan-3 expression positively correlated with a macrophage gene signature across several TCGA cancer types. *In vitro* experiments demonstrated that hypoxia (1% oxygen) or treatment with IFN-γ stimulate Sdc-3 expression on RAW-264.7 derived macrophages, linking Sdc-3 expression to a proinflammatory response. Syndecan-3 expression correlates with a better patient overall survival in hypoxic melanoma tumors.

## Introduction

In the last decade, syndecans (Sdc) have attracted attention in cancer research since they are markedly dysregulated in the tumor microenvironment (TME) (1–4). Syndecans are an evolutionary conserved family of small type I transmembrane proteoglycans that are involved in the organization and assembly of extracellular matrix (ECM) (5, 6). They consist of a protein core to which heparan sulfate chains are covalently attached. The Sdc family is comprised of four members of cell surface molecules (Sdc-1 to 4) with different biological functions in development, health and disease. They can act as receptors for cytokines, chemokines, morphogens, and growth factors to regulate different signaling pathways (7, 8). Thereby Sdc can affect many physiological and pathological processes, including cancer and immunity (5, 9–11).

Syndecans interact with integrins and growth factor receptors to modulate cell signaling and the ECM, potentially having an impact on tumor progression as suggested by previous studies focused on Sdc-1, Sdc-2, and Sdc-4 (12).

Syndecan-3 is known to be mainly expressed in the brain and nervous tissue and plays a key role in cell adhesion and migration (13, 14). However, its expression is not restricted to neuronal tissues, and recent studies point to important roles of Sdc-3 in inflammatory disease, regulation of energy balance, cancer, angiogenesis, and viral infections (12, 14–22). There is some evidence that Sdc-3 is expressed in the TME and several cancer cell lines, including bladder cancer (23), hepatocellular carcinoma (22, 24), mammary carcinoma (25), ovarian cancer (19, 20), pancreatic cancer (26), prostate carcinoma (27), renal cell carcinoma (12, 28), and several glioma cell lines (29). However, the patterns of expression and significance of Sdc-3 in cancer and infiltrating immune cells remains unexplored.

A common feature of all types of solid tumors is an imbalance between cancer cell proliferation and blood supply, which results in hypoxia. The hypoxic response in cells is mediated by the family of hypoxia inducible factor (HIF) transcription factors, which play an integral role in the metabolic changes that drive cellular adaptation to low oxygen availability. In response to hypoxia, a large number of target genes involved in cell growth, metabolism, metastasis, and immunity are activated in cancer cells (30–32). Hypoxia also modulates the fate and function of different immune populations, including T cells and macrophages (33), and the organization of the ECM that influences their migration and infiltration (34).

Macrophages are abundant in the TME and preferentially accumulate within tumor hypoxic regions (35). Due to their plasticity, they can polarize into pro-inflammatory and anti-tumor or into anti-inflammatory and pro-tumor agents. Thus, the interaction between cancer cells and tumor-associated macrophages (TAMs) within the hypoxic TME is one of the major features that can dictate the tumor malignancy and progression (36). Indeed, their infiltration has been associated with poor prognosis and therapy resistance in several malignancies (37).

Current state-of-the-art immunotherapies are only effective in a fraction of patients, and several combinatorial approaches have recently failed in the clinic, resulting in an urgent medical need in several cancer types (38). In this context, further knowledge on Sdc-3 regulation and its biological significance in the TME could contribute to the development of new immunotherapies that may widen the spectrum of patients who benefit from these treatments.

In this study, we have found that expression of Sdc-3 is dysregulated in several cancer types and expressed in cancer cells and macrophages, as a result of limited oxygenation in the TME. Syndecan-3 expression correlates with markers of hot tumors such as gamma interferon (IFN-γ), and its expression can predict a better patient overall survival (OS).

## Materials and Methods

### Bioinformatic Analyses

#### Gene Expression on Human Tumors

We selected 21 tumors from The Cancer Genome Atlas program (TCGA) database presenting a significant dysregulation on the expression of Sdc-3 or Sdc-4 between malignant versus normal or adjacent tissue on Gepia 2.^1^ The normalized raw data for the gene expression on each tumor and associated normal tissue (TCGA TARGET GTEx dataset) was downloaded through the UCSC Xena browser (39) in log_2_(norm_count + 1) format. The number of tumors and normal or adjacent tissue samples considered in each cancer type are described in Supplementary Table 1. Statistical analyses were performed using GraphPad Prism version 8.0.

#### Expression of *SDC3* or *SDC4* Genes on Human Cell Lines

We obtained the normalized gene expression for these two genes from Iorio et al. (40). A total of 1,018 human cell lines were ordered by the expression level of *SDC3* or *SDC4* genes. We selected and plotted the top 100 cell lines expressing the highest levels of *SDC3* or *SDC4* in each case, grouped by representative tissue origin.

#### *SDC3* and *SDC4* Gene Expression Across Human Normal Tissues and Stromal Cells

Data corresponding to normal tissue and stromal cells (mean and SD) were collected from the GeneAtlas U133A (41) and Primary Cell Atlas (42) datasets, respectively, through the BioGPS portal.^2^

#### Correlations Between *SDC3* or *SDC4* and Gene Signatures

Spearman correlation analyses between *SDC3* or *SDC4* and the two different gene signatures used in this study were performed in Gepia 2. The hypoxia signature was defined by Ye et al. (43) and includes the following genes: *ACOT7*, *ADM*, *ALDOA*, *CDKN3*, *ENO1*, *LDHA*, *MIF*, *MRPS17*, *NDRG1*, *P4HA1*, *PGAM1*, *SLC2A1*, *TPI1*, *TUBB6*, and *VEGFA*). The macrophage signature consisted of a set of 92 genes defined by Tirosh et al. (44).

#### Correlations Between *SDC3* or *SDC4* and Other Genes

We performed Spearman and Pearson correlation analyses between *SDC3* or *SDC4* and other genes (*IL4*, *IFNG*, *CD274*, *CD8A*, or *HIF1A*). For that purpose, we downloaded the normalized RSEM log_2_(*x* + 1) data for each TCGA cohort analyzed through the UCSC Xena browser.

#### Evaluation of *SDC3* and *SDC4* Gene Expression in the TME

Single-cell RNA sequencing data (scRNA-seq) of 19 melanoma tumors were obtained from Tirosh et al. (44). Raw data were processed by SeqGeq version 1.6.0 (FlowJo) and normalized to counts per million (CPMs). We used the cell type specific gene-signatures provided by the authors to cluster the immune and tumor cell populations in the TME. We assessed the relative expression of *SDC3* and *SDC4* within each stromal and tumor cell population, including B cells, cancer associated fibroblasts (CAFs), CD4^+^ and CD8^+^ T cells, endothelial cells, tumor associated macrophages (TAMs), and malignant cells.

### Cell Culture

Cell lines were obtained from the American Type Culture Collection (ATCC) and cultured according to standard mammalian tissue culture protocols and sterile technique. The murine RAW-264.7 macrophages (ATCC #TIB-71) were cultured in DMEM (Gibco #41966), CT26 cells from mice (ATCC #CRL-2638) were cultured in RPMI medium 1640 GlutaMAX (Gibco #61870044). All media was supplemented with 10% FBS and 1% Penicillin-Streptomycin. All cells were tested for mycoplasma before performing the experiments using the MycoAlert mycoplasma detection kit (Lonza #LT27-221) following manufacturer’s instructions. For M1 polarization of RAW-264.7 cells, 300.000 cells/well were seeded in a 6-well plate with 100 ng/mL of IFN-γ (Biolegend #575304) and for M2 polarization, cells were co-cultured with 100 ng/mL of IL-4 (Biolegend #574304). Hypoxia cultures were performed at 1% oxygen and 5% CO_2_ in an In Vivo2 400 hypoxic station (Ruskinn Technologies).

### Knockout of *HIF1A* Gene in CT26 Cell Line via CRISPR/Cas9

We generated HIF-1α knockout CT26 murine cells (HIF1-KO) using TrueGuide CRISPR single guide RNAs (sgRNAs) targeting the first exon of the *Hif1a* gene (sgRNA sequence: uuucuucucguucucgccgc) and the Cas9 nuclease 2NLS (Synthego). RNP-complexes (1.3:1 sgRNA to Cas9 ratio) were introduced in the cells using Lipofectamine CRISPRMAX Cas9 Transfection reagent (ThermoFisher #CMAX00001) and following the *CRISPR Editing of Immortalized Cell Lines with RNPs Using Lipofection* protocol by Synthego. Transfected cells were cultured in Opti-MEM Reduced Serum Medium (ThermoFisher #31985062) for eight hours and then in RPMI 1640 Medium GlutaMAX (ThermoFisher #61870044) supplemented with 10% FBS and 1% Penicillin-Streptomycin. Seventy-two hours after transfection a limiting dilution (1 cell/mL) was carried out in a 96-well plate followed by clonal expansion. The knockout of *Hif1a* was checked by Sanger DNA sequencing (StabVida) using primers flanking the sgRNA target region. The HIF1-KO presented a 72 bp deletion affecting the first seven aminoacids of the protein. The deletion was confirmed by quantitative PCR (qPCR) using primers that hybridize within the affected 72 bp region to check the absence of amplification (Supplementary Table 2).

### Quantitative PCR

Total RNA was purified using the NucleoSpin RNA kit (Macherey-Nagel #740955.250), diluted in 50 μL of molecular grade water, and quantified using a NanoDrop ND-1000 spectrophotometer (Thermo Fisher Scientific). cDNA was synthesized by reverse transcription from 250 nG-1μG of purified RNA with the M-MLV Reverse Transcriptase (Thermo Fisher Scientific #28025-013) and Random Primers (Thermo Fisher Scientific #58875) in a final reaction volume of 20 μL. The qPCR reactions were conducted in triplicate on a ViiA 7 Real-Time PCR System (Thermo Fisher Scientific) from 1 μL of cDNA using the PerfeCTa SYBR Green SuperMix reagent (Quantabio #95056-500) and gene-specific primers (Supplementary Table 2). After an initial denaturation at 95°C for 3 min, samples were subjected to 40 cycles of denaturation at 95°C for 15 s, annealing at 60°C for 60 s, and extension at 72°C for 60 s. Data were analyzed using the QuantStudio software version 1.3 (Thermo Fisher Scientific). The relative quantification in gene expression was determined using the 2^–ΔΔCt^ method. The *Rplp0* gene (also known as *36b4*), coding for the ribosomal protein large P0, was used as an internal control to normalize the data.

### Flow Cytometry

Cells were collected and stained with the LIVE/DEAD Fixable Blue Dead Cell Stain kit (Thermo Fisher Scientific #L23105) for 30 min at 4°C. After that, cells were washed (600 × *g* for 5 min) and incubated with the eBioscience Flow Cytometry staining buffer (Thermo Fisher Scientific #00-4222-26) containing anti-Sdc-3 (Thermo Fisher Scientific #PA5-100116) antibodies (1:200). Cells were washed, incubated with an anti-rabbit Alexa Fluor 647 (Thermo Fisher Scientific #A-21245) secondary antibody (1:200) for 30 min at 4°C, washed again and resuspended in 200 μL of staining buffer. Cells were acquired on a FACSymphony cytometer (BD Biosciences) and results were analyzed using FlowJo version 10 (BD Biosciences).

### Western Blot

Nuclear and cytoplasmic protein fractions were isolated using the NE-PER Nuclear and Cytoplasmic Extraction Reagents (Thermo Fisher Scientific #78835). Protein quantification was performed using the BCA Protein Assay Kit (Thermo Fisher Scientific #23227). Samples were mixed with NuPAGE LDS Sample Buffer (4×) (Invitrogen #NP0007) containing DTT and heated for 15 min at 95°C. Each preparation was separated in a 4–15% Mini-PROTEAN TGX Precast Protein Gel (BioRad #4561083) with 1X Tris/Glycine/SDS electrophoresis buffer (BioRad #1610772). PageRuler Plus Pretrained Protein Ladder (Thermo Fisher Scientific #26619) was used to calculate the molecular weight of the proteins. The proteins were transferred to a 0.2 μm PVDF membrane (BioRad #1704156) using a Trans-Blot Turbo Transfer System (BioRad) and blocked for 1 h in 5% skim milk (Millipore #70166) and 0.5% Tween-20 (Sigma Aldrich #P2287) diluted in PBS (Fisher BioReagents #BP3994). Then, primary antibodies were added and incubated overnight, followed by five washes with PBS (containing 0.5% Tween-20) and incubation with secondary HRP-conjugated antibodies (1:5000). After the incubation with the secondary antibody five additional washes were carried out. Primary antibodies against HIF-1α (1:500) (Novus Biologicals #NB100-449), the nuclear matrix protein p84 (1:5000) (Abcam #487) and β-tubulin (1:5000) (ThermoFisher #MA5-16308) were used. HRP-conjugated anti-Mo (#S301677076S) and anti-Rb (#S301677074S) antibodies were obtained from Cell Signaling. Chemiluminescence detection was performed using Clarity Max Western ECL Substrate (BioRad #170506) on an iBright CL1500 system (Invitrogen).

## Results

### Expression of Sdc-3 and Sdc-4 Is Significantly Dysregulated in Several Cancer Types

**FIGURE 1:**
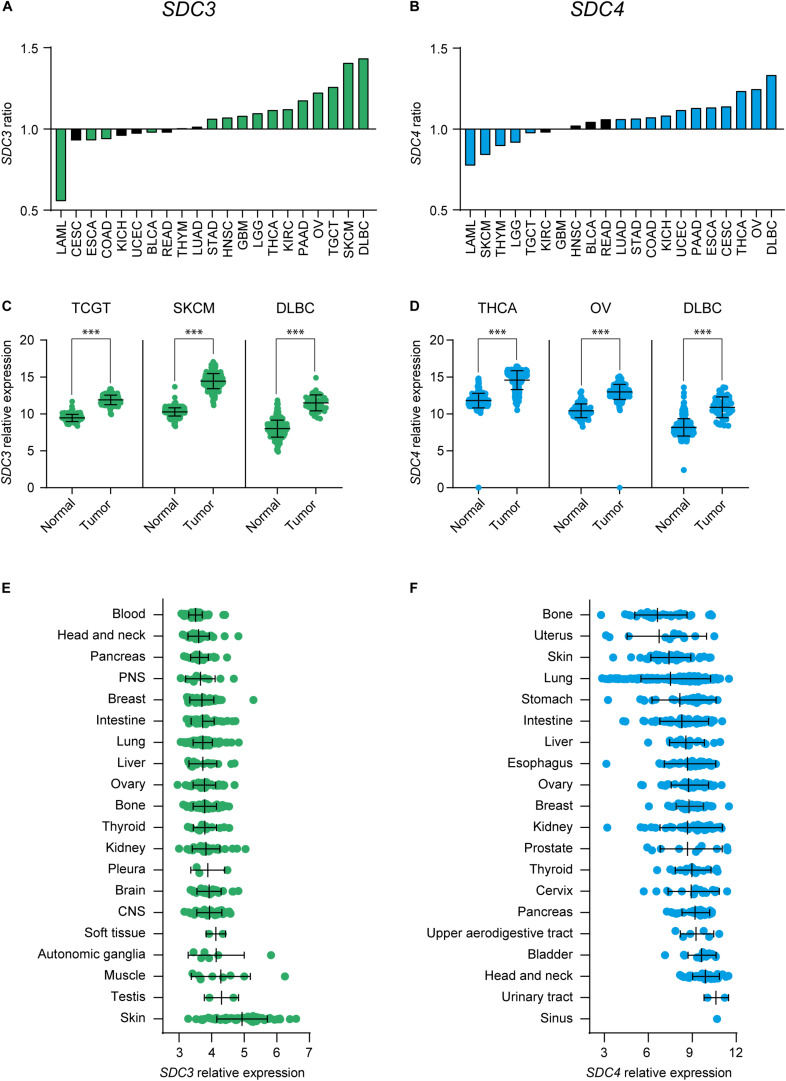
Expression of Sdc-3 and Sdc-4 is significantly dysregulated in several cancer types. **(A,B)** Ratio (y axis) of expression of *SDC3* (A) or *SDC4* (B) genes on malignant versus normal or adjacent tissue. Tumor types with a significant difference of expression are shown. **(C,D)** Dot plots showing the normalized expression of *SDC3* (C) or *SDC4* (D) in the three tumor types that shown the most significant upregulation in each case. Asterisks represent p values of unpaired T test (***, < 0.001). **(E,F)** Mean RNA expression level (normalized) of Sdc-3 (E) and Sdc-4 (F) across several human cell lines representative of different tissue origin. CNS, Central Nervous System; PNS, Peripheral Nervous System.

First, we checked *SDC3* and *SDC4* gene expression levels on malignant and healthy tissue on several types of cancer on the TCGA database (Supplementary Table 1). Figure 1 shows the tumor types that exhibited a significant upregulation, and to lower extent downregulation, of Sdc-3 (Figure 1A) and Sdc-4 (Figure 1B) levels based on the ratio of expression on malignant versus normal or adjacent tissue. Focusing on the different tumor types, testicular germ cell tumors (TGCT), skin cutaneous melanoma (SKCM), and diffuse large B-cell lymphoma (DLBC) shown the highest ratio for Sdc-3 (Figure 1C), while thyroid carcinoma (THCA), ovarian serous cystadenocarcinoma (OV), and DLBC were the highest for Sdc-4 (Figure 1D). We also studied the levels of expression of Sdc-3 and Sdc-4 on human tumor cell lines representative of different tissue origin from a publicly available dataset (40). In general, the levels of expression of Sdc-4 were higher than Sdc-3 (Figures 1E,F). Syndecan-4 (Sdc-4) expression was also higher than that of Sdc-3 in healthy tissues analyzed from the BioGPS database (Supplementary Figure 1). This analysis also shown that the expression of Sdc-3 (Supplementary Figure 1A) on healthy tissue is confined to spinal cord and brain, as previously described (13, 14), while the expression of Sdc-4 (Supplementary Figure 1B) is broader. In a similar fashion to healthy tissue, the expression of Sdc-4 was more homogeneous across the different malignant cell lines (Figure 1F), while the expression of Sdc-3 was mostly enriched in skin cutaneous melanoma (SKCM) cell lines (Figure 1E), in contrast to its low expression in normal skin (Supplementary Figure 1A).

### Expression of Sdc-3 and Sdc-4 Correlates With a Hypoxia Signature in Several Cancer Types

**FIGURE 2:**
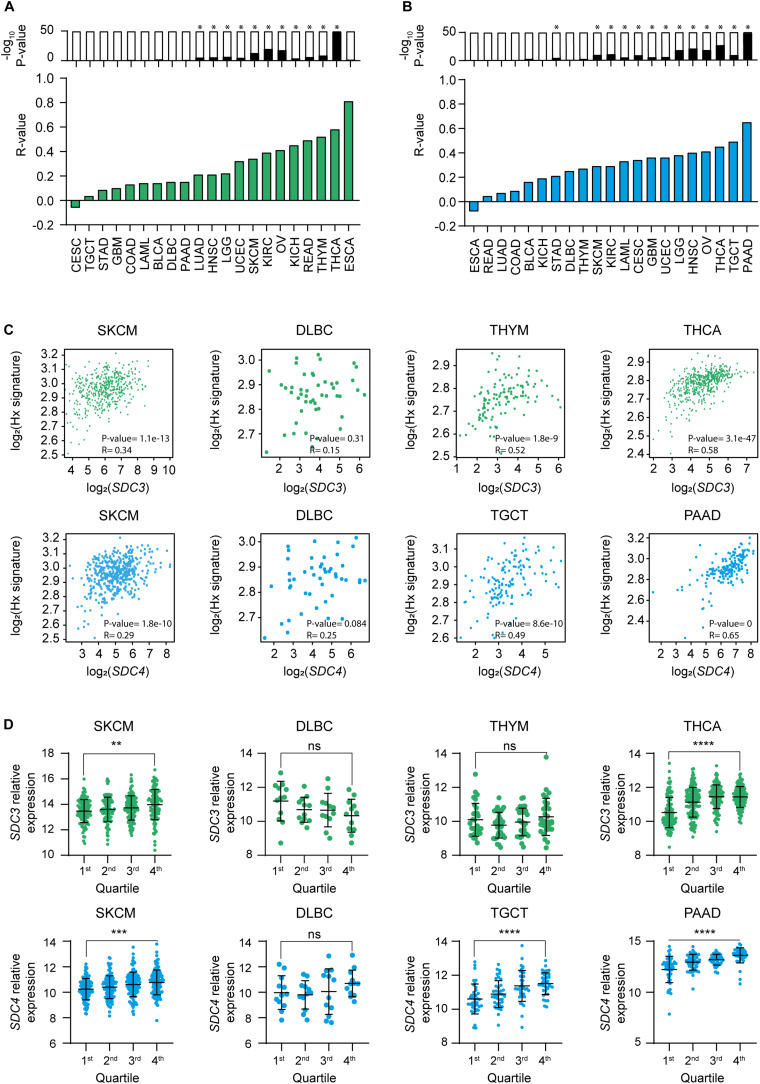
Expression of Sdc-3 and Sdc-4 correlates with a hypoxia signature in several cancer types. **(A,B)** Spearman correlation analyses between *SDC3* (A) or *SDC4* (B) genes and a hypoxia gene signature. Green and blue bars represent r values. P values are represented by bars on top (−log_10_ scale), significant *p* values (*p* < 0.001) are indicated with an asterisk (*). **(C)** Dot plots showing the correlation (Spearman) between *SDC3* (upper panels, green) or *SDC4* (lower panels, blue) genes and the hypoxia signature genes for SKCM and DLBC, as well as the top two cancer types ranked in panels **(A,B)** in each case. Both *r* and *p* values are shown. **(D)** Dot plots showing the normalized expression of *SDC3* (upper panel, green) or *SDC4* (down panel, blue) for the indicated tumor types. Each cohort was divided in four quartiles (Q1–Q4) according to the normalized level of expression of the hypoxia signature genes. P values of the one-way ANOVA test are shown for the Q1 and Q4 comparison. Asterisks represent P values for each statistical test as follows: ns (*p* > 0.05), *(*p* ≤ 0.05), **(*p* ≤ 0.01), ***(*p* ≤ 0.001), and ****(*p* ≤ 0.0001).

Given that the majority of cancer types that shown a significant positive ratio of expression of Sdc-3 and Sdc-4 on malignant versus normal or adjacent tissue are solid tumors (Figures 1A,B), and solid tumors are known to be hypoxic, we interrogated the data for potential correlations between *SDC3* or *SDC4* genes and a hypoxia signature comprised of 15 genes including *LDHA*, *VEGFA*, and others (see section “Materials and Methods”). We found that *SDC3* (Figure 2A) and *SDC4* (Figure 2B) positively correlated with the expression of the hypoxia signature on several solid tumor types, based on an analysis of the TCGA database. Skin melanoma, the solid tumor type with the highest ratio of expression of Sdc-3 on malignant versus normal tissue (Figures 1A,C), shown a positive correlation with the hypoxia signature for both *SDC3* (Figures 2A,C) and *SDC4* (Figures 2B,C), while DLBC, a non-solid tumor, presented no significant correlation (Figures 2A–C). Other tumor types with a significant correlation with the hypoxia signature were thymoma (THYM) and THCA for *SDC3*, and TGCT and pancreatic adenocarcinoma (PAAD) for *SDC4* (Figure 2C). To further explore their expression patterns on hypoxic tumors, we stratified the data on quartiles according to the normalized level of expression of the hypoxia gene signature. As can be seen in Figure 2D, Sdc-3 expression increased with hypoxia levels in SKCM and THCA, and Sdc-4 in SKCM, TGCT, and PAAD.

### Sdc-3 and Sdc-4 Are Expressed in Tumor and Stromal Cell Populations in the TME

**FIGURE 3:**
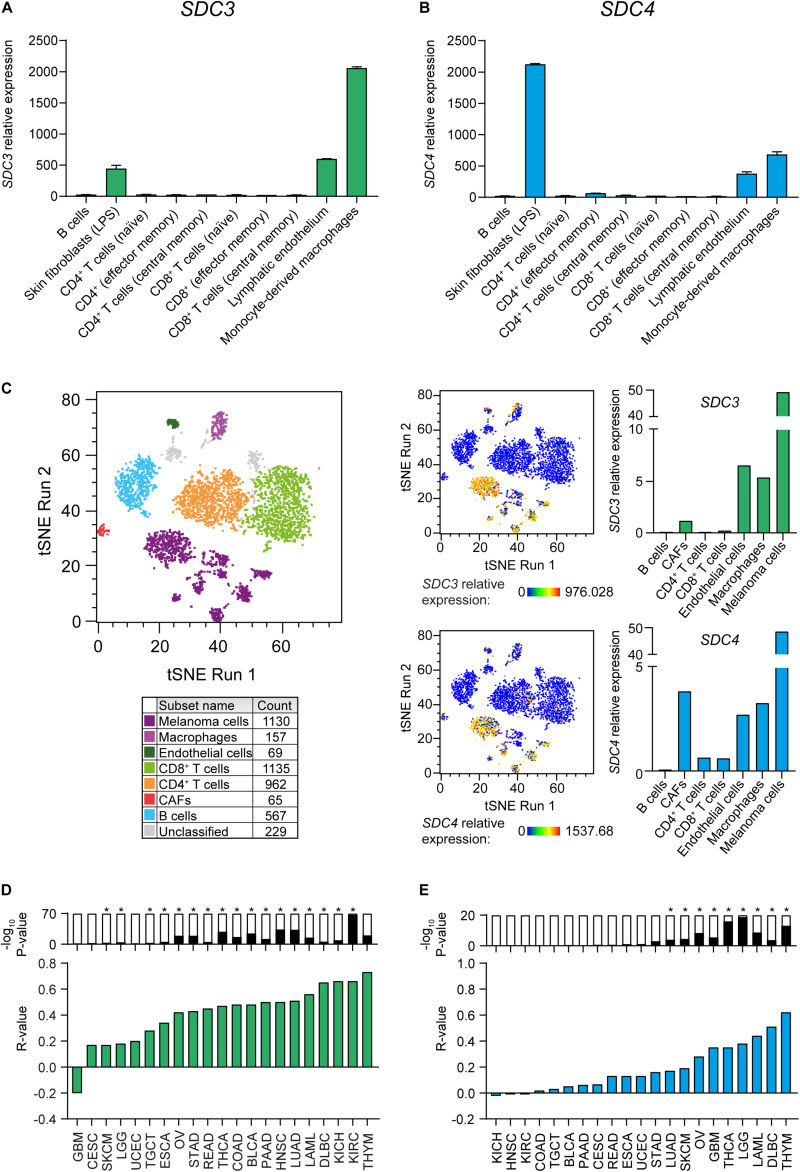
Sdc-3 and Sdc-4 are expressed in tumor and stromal cell populations in the TME. **(A,B)** Relative expression of *SDC3* **(A)** and *SDC4* **(B)** genes across different stromal cell populations. Data (mean and SD) were extracted from the Primary Cell Atlas dataset through BioGPS. **(C)** T-SNE plots showing the expression of Sdc-3 and Sdc-4 on several stromal cell populations in the TME from human melanoma tumors based on scRNA-seq data analysis. Cell populations were defined by cell-type specific gene signatures. **(D)** Spearman correlation analyses between *SDC3* **(D)** or *SDC4* **(E)** genes and a macrophage gene signature. Green and blue bars represent r values. P values are represented by bars on top (−log_10_ scale), significant P values (*p* < 0.001) are indicated with an asterisk (*).

We investigated which stromal cell types express Sdc-3 and Sdc-4 by examining the Primary Cell Atlas dataset on BioGPS. Syndecan-3 was mostly expressed by monocyte-derived macrophages, and to lower extent on lymphatic endothelium cells and skin fibroblasts (Figure 3A). Syndecan-4 shown a similar pattern, but with higher expression on skin fibroblasts than that of Sdc-3 (Figure 3B). No Sdc-3 or Sdc-4 gene expression was detected on B cells or any type of CD4+ or CD8+ T cells (naïve, effector, and central memory) (Figures 3A,B). In order to examine the expression of Sdc-3 and Sdc-4 in the TME, we analyzed single-cell RNA sequencing (scRNA-seq) data from melanoma patients, which was the solid tumor type with the highest ratio of expression of Sdc-3 on malignant/normal tissue based on TCGA database. We defined malignant and stromal components of the TME by established gene signatures to characterize tumor cells, TAMs, CD4+ and CD8+ T cells, B cells, and CAFs (Figure 3C). *SDC3* and *SDC4* genes expression levels were the highest on melanoma cells and were also present on macrophages and endothelial cells but absent on B and T cells. Syndecan-4 expression was higher on CAFs than that of Sdc-3 (Figure 3C). Together, the expression patterns found in the melanoma TME by scRNA-seq are in accordance with those from the Primary Cell Atlas dataset (Figures 3A,B). Interestingly, the expression of Sdc-3 on TAMs was higher than the expression of Sdc-4. To further explore this finding in other cancer types, we performed correlations between a macrophage gene signature panel comprised of 92 genes and *SDC3* (Figure 3D) or *SDC4* (Figure 3E) genes based on TCGA database. We found a significant positive correlation between the macrophage gene signature and both genes across different tumor types, especially for Sdc-3.

### Hypoxia Upregulates Sdc-3 Expression on Tumor Cells on a HIF-1α Dependent Mechanism

**FIGURE 4:**
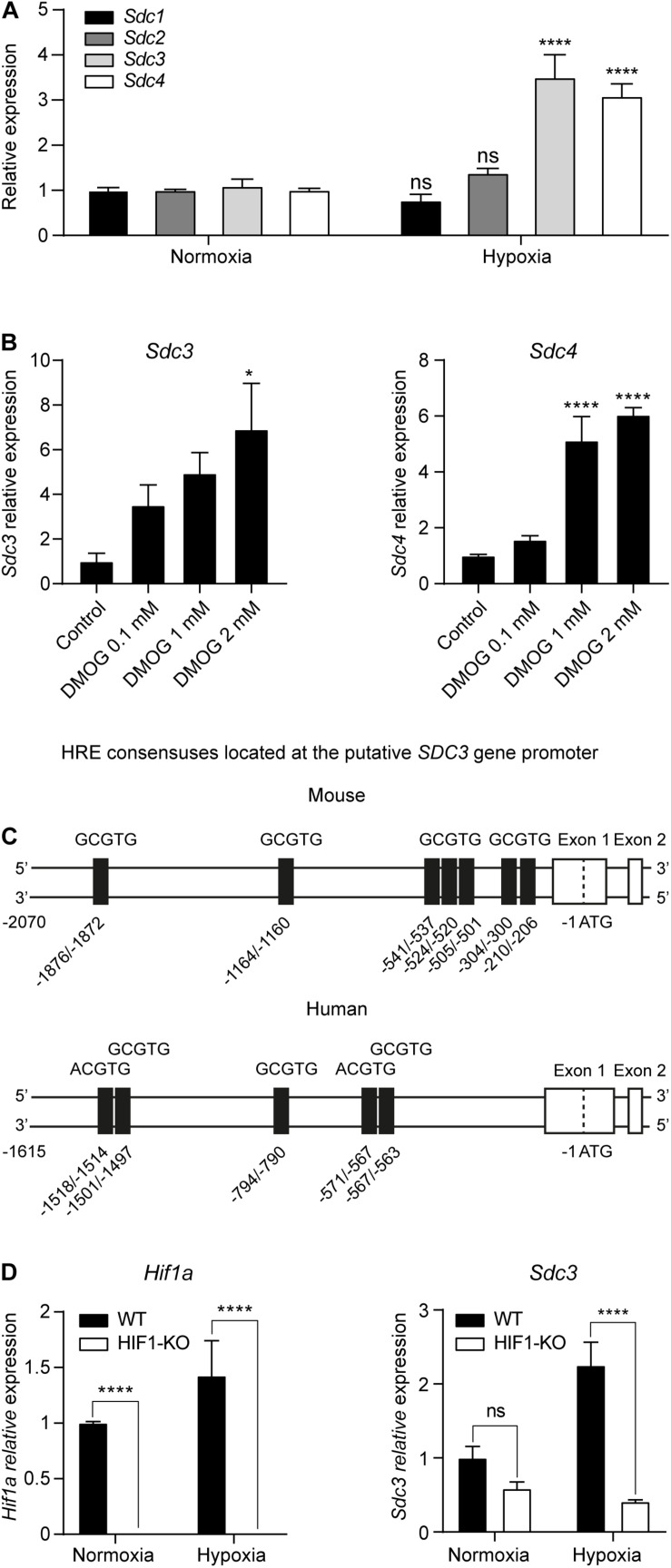
Hypoxia upregulates Sdc-3 expression on tumor cells on a HIF-1α dependent mechanism. **(A)** Expression of Sdc-1, Sdc-2, Sdc-3, and Sdc-4 on mouse tumor CT26 cells cultured under normoxia (21% oxygen) or hypoxia (1% oxygen) for 24 h. A pool of three independent experiments is shown, error bars represent SEM (unpaired T test). **(B)** Expression of Sdc-3 (left) and Sdc-4 (right) on CT26 cells treated with increasing doses of DMOG (0, 0.1, 1, and 2 mM) under normoxia for 48 h. One representative experiment is shown, error bars represent SD (one-way ANOVA test). **(C)** Schematic linear map of HREs (5′-RCGTG-3′, being R: A or G) in the predicted mouse (upper) and human (lower) putative promoter of Sdc-3. **(D)** Expression of *Hif1a* (left panel) and *Sdc3* (right) genes on WT and HIF1-KO CT26 cells cultured under normoxia or hypoxia for 24 h. A pool of two independent experiments is shown, error bars represent SEM (two-way ANOVA test). Asterisks represent p values for each statistical test as follows: ns (*p* > 0.05), * (*p* ≤ 0.05), ** (*p* ≤ 0.01), *** (*p* ≤ 0.001), and **** (*p* ≤ 0.0001).

In order to study the influence of oxygenation on the expression of Sdc on malignant cells, we cultured the mouse tumor cell line CT26 under normoxia (21% oxygen) or hypoxia (1% oxygen) for 24 h and performed a qPCR. Figure 4A shows that hypoxia induced the transcription of both *Sdc3* and *Sdc4* genes. We then checked if the HIF molecular pathway was responsible for the observed phenomenon by culturing CT26 tumor cells under normoxia with increasing doses of dimethyloxalylglycine (DMOG), a chemical inhibitor of prolyl and asparaginyl hydroxylases. DMOG prevents HIF-1α degradation, leading to its stabilization and an increase of its transcriptional activity. Figure 4B shows that treatment with DMOG increased the expression of Sdc-3 (left panel) and Sdc-4 (right panel) on a dose dependent manner, suggesting that HIF is involved in the transcriptional control of both genes under hypoxia. To further characterize this response, we examined the putative mouse and human promoters of Sdc-3 where we found several predicted hypoxia response elements (HREs) (Figure 4C). This pointed to a potential role of HIF on the direct regulation of Sdc-3 expression under hypoxia. To confirm this hypothesis, we generated a HIF1-KO via CRISPR/Cas9. The knockout clone presented a 72 bp frameshift deletion that was revealed by Sanger sequencing (Supplementary Figure 2A) and qPCR using primers targeting the deleted region (Figure 4D, left panel). HIF-1α knockout CT26 cells failed to upregulate known HIF-1α target genes (*Pgk1* and *Vegfa*) when subjected to hypoxia culture (Supplementary Figure 2B), confirming that our knockout model lacks functional HIF-1α protein. Accordingly, HIF1-KO tumor cells did not present HIF-1α protein in the nucleus after 4 h incubation under hypoxia (Supplementary Figure 2C), demonstrating that the generated cell line is suitable for the interrogation of the potential role of HIF-1α in the hypoxic upregulation of Sdc-3. As can be seen on Figure 4D (right panel), HIF1-KO tumor cells failed to upregulate the expression of Sdc-3 under hypoxia as opposed to the wild type (WT) tumor cells.

### Hypoxia and IFN-γ Drives Sdc-3 Expression on Tumor Associated Macrophages

**FIGURE 5:**
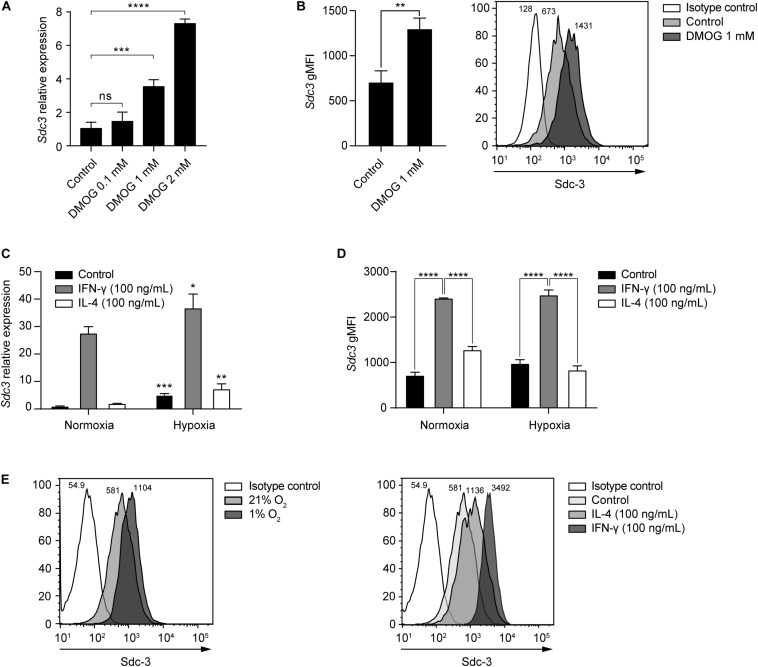
Hypoxia and IFN-γ drive Sdc-3 expression on tumor associated macrophages. **(A)** Relative gene expression of Sdc-3 on macrophages derived from murine RAW-264.7 cells treated with increasing doses of DMOG (0, 0.1, 1, and 2 mM) under normoxia (21% oxygen) for 24 h. One representative experiment is shown, error bars represent SD (one-way ANOVA test). **(B)** Sdc-3 protein expression on macrophages derived from RAW-264.7 cells treated with 1 mM DMOG or vehicle control measured by flow cytometry. One representative experiment is shown, error bars represent SD (unpaired T test), *n* = 3. A representative histogram showing the geometric Mean Fluorescent Intensity (gMFI) values for each peak is also presented. **(C)** Relative gene expression of Sdc-3 on macrophages treated with IFN-γ (100 ng/mL) or IL-4 (100 ng/mL) under normoxia or hypoxia (1% oxygen). One representative experiment is shown, error bars represent SD. Asterisks indicate the p values of the unpaired T test between each condition (normoxia vs. hypoxia). **(D)** Flow cytometry histograms showing the expression of Sdc-3 protein on macrophages treated with IFN-γ (100 ng/mL) or IL-4 (100 ng/mL) under normoxia or hypoxia. A pool of two independent experiments is shown, error bars represent SEM (two-way ANOVA test). **(E)** Representative flow cytometry histograms of Sdc-3 expression on macrophages under normoxia or hypoxia (left) or treated with IFN-γ (100 ng/mL) or IL-4 (100 ng/mL) under normoxia (right); gMFI values corresponding to an independent experiment from panel **(D)** are shown. Asterisks represent P values for each statistical test as follows: ns (*p* > 0.05), * (*p* ≤ 0.05), ** (*p* ≤ 0.01), *** (*p* ≤ 0.001), and **** (*p* ≤ 0.0001).

In addition to tumor cells, we identified TAMs as the main stromal compartment expressing Sdc-3 in the TME based on scRNA-seq data from melanoma patients (Figure 3C). We interrogated macrophages derived from murine RAW-264.7 cells *in vitro* for Sdc-3 gene and protein expression when cultured in the presence of increasing doses of DMOG or under normoxia (21% oxygen) *versus* hypoxia (1% oxygen) (Figures 5A–E). Both increasing doses of DMOG and hypoxia induced the expression of Sdc-3 on macrophages at the RNA (Figures 5A,C, respectively) and protein (Figures 5B,D, respectively) levels. We also checked the influence of pro-inflammatory (IFN-γ) and anti-inflammatory (IL-4) cytokines on the expression of *Sdc3* gene (Figure 5C). Gamma interferon, but not IL-4, induced a strong upregulation of Sdc-3 under normoxia (27-fold) and hypoxia (36-fold) at RNA level (Figure 5C). This upregulation was confirmed at the protein level by flow cytometry (Figures 5D,E). These findings are further supported by the fact that IFN-γ, but not IL-4, induces the stabilization of HIF-1α at the protein level, as previously described by Takeda et al. (45). Moreover, Sdc-3 positively correlated with the expression of *IFNG* but not *IL4* genes across the majority of TCGA tumors analyzed (Supplementary Table 3), as opposed to Sdc-4 that did not shown a broad correlation (Supplementary Table 4). Syndecan-3 also correlated with genes related to the IFN-γ pathway, such as *CD274* and *CD8A*.

### Sdc-3 Expression Correlates With a Better Overall Survival on Hypoxic Melanoma Tumors

**FIGURE 6:**
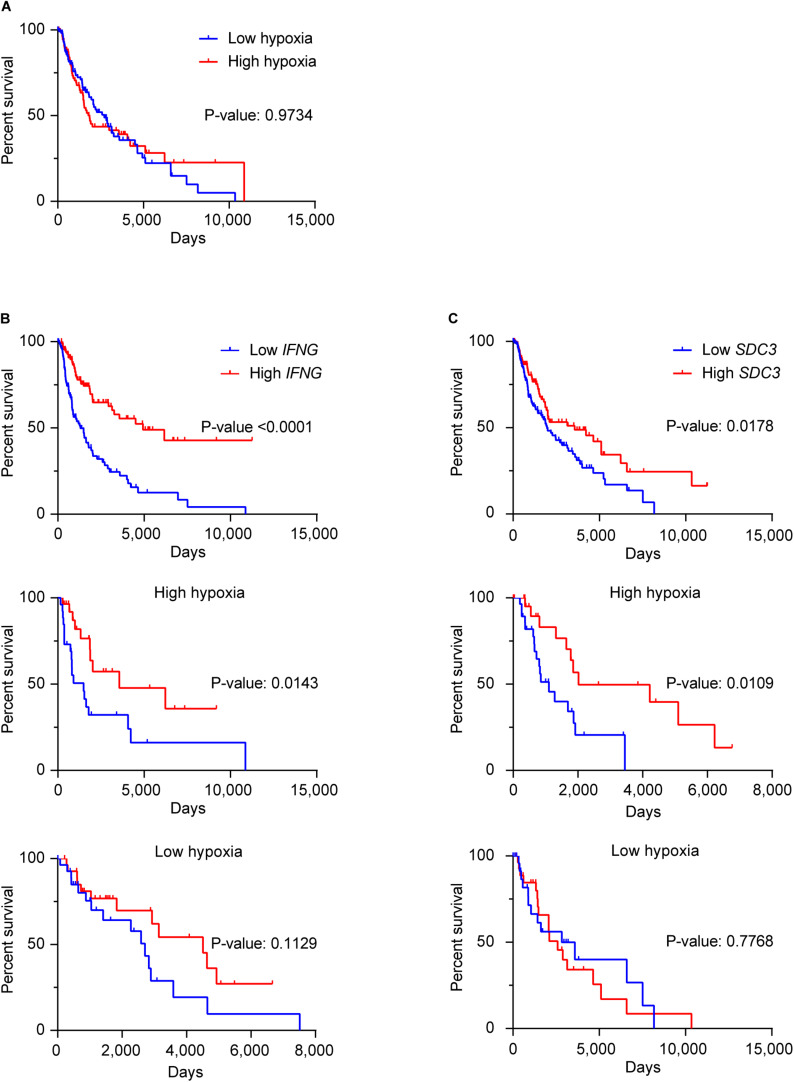
Sdc-3 expression correlates with a better overall survival on hypoxic melanoma tumors. **(A)** Kaplan-Meier estimates of OS among skin melanoma patients (from the TCGA-SKCM cohort) showing low or high hypoxia profiles. **(B,C)** Kaplan–Meier analysis of OS among patients with normalized low or high expression of *IFNG* **(B)** or *SDC3* **(C)** genes in the general cohort (upper panel), as well as the patients exhibiting high (central panel) or low (lower panel) hypoxic tumors. The SKCM cohort was split in four quartiles (from Q1 to Q4) according to the normalized level of expression of the hypoxia gene signature **(A)**, *IFNG* **(B)**, or *SDC3* **(C)** in each case. Lower (Q1) and upper (Q4) quartiles are depicted, and P values are shown (log-rank or Mantel–Cox test).

We investigated the potential prognostic value of Sdc-3 expression on SKCM patients by analyzing OS data on the TCGA database. Figure 6A shows that different levels of expression of the hypoxia gene signature does not impact patient OS. High expression of *IFNG* gene predicts a significant increase of OS (Figure 6B). Interestingly, when stratifying the cohort based on the high or low expression of the hypoxia signature genes, the increase of the OS based on *IFNG* gene expression was only significant on the most hypoxic tumors (Figure 6B). On a similar fashion, high Sdc-3 expression significantly correlated with a better OS in melanoma (Figure 6C). This difference was only present in highly hypoxic tumors, suggesting that Sdc-3 expression is associated with hypoxia and a proinflammatory immune response, leading to better patient OS in melanoma.

## Discussion

**FIGURE 7:**
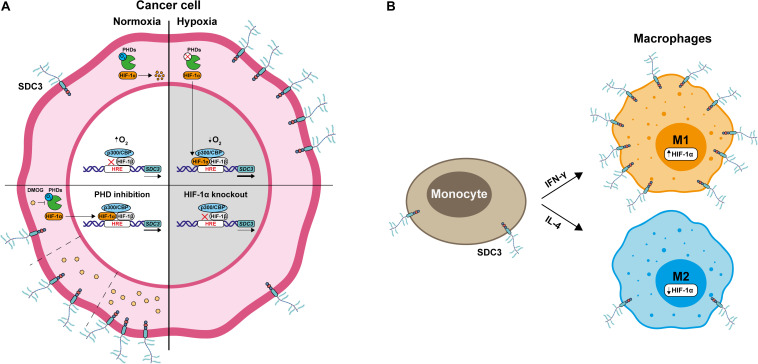
HIF-1α promotes Sdc-3 expression. **(A)** Under normoxia, cancer cells present low expression of Sdc-3. When oxygen is available, prolyl-hydroxylases (PHDs) are active and mark HIF-1α for proteasomal degradation, preventing its translocation to the nucleus. In contrast, a shortage of oxygen results in the inhibition of PHDs and accumulation of HIF-1α, which translocates to the nucleus to bind to HREs and promote Sdc-3 expression. Treatment with increasing doses of DMOG, a PHD inhibitor, also results in increased Sdc-3 expression. This suggests that HIF-1α is responsible for the hypoxic up-regulation of Sdc-3. This hypothesis was confirmed with a HIF1-KO cell line that failed to upregulate Sdc-3 under hypoxia. **(B)** M1-like macrophages derived from monocytes after treatment with IFN-γ present higher levels of expression of Sdc-3 than M2-like macrophages treated with IL-4.

Here we show for the first time that hypoxia induces Sdc-3 expression on a HIF-1α dependent mechanism. Syndecan-3 is expressed on tumor cells, macrophages, and endothelial cells in the TME (Figure 7). These results suggest that Sdc-3 may have a functional role in solid tumors with limited oxygen availability. Another member of the Sdc family, Sdc-4, has previously been described as a HIF-1α target in other cell types (46). Here we also show that Sdc-4 correlates with a hypoxia signature and gets upregulated on tumor cells upon inhibition of PHDs. The pattern of expression of Sdc-4 in tumor infiltrating cells is similar to that of Sdc-3, with the exception of a higher expression on CAFs. On the contrary, we have not observed that Sdc-1 or Sdc-2 expression is controlled by hypoxia.

We found a broad upregulation of the expression of Sdc-3 in several tumor types. Because solid tumors are known to be hypoxic, we tested the hypothesis if the expression of Sdc-3 was influenced by hypoxia. Experiments performed with DMOG demonstrated that the inhibition of PHDs directly control the expression of Sdc-3, and we confirmed this finding with a HIF-1α deficient tumor cell line, and by the identification of several HREs on the Sdc-3 promoter. Taken together, our results indicate that Sdc-3 expression is hypoxia-sensitive and depends on HIF-1α activity in tumor cells.

Next, we investigated the expression of Sdc-3 in other cell types present in the TME. Apart from cancer cells, endothelial cells and macrophages were identified as Sdc-3 expressing cell populations. Expression of Sdc-3 by endothelial cells has been previously reported (21) in the context of inflammatory disease (47) but its potential role on tumor angiogenesis is still largely unexplored (14). Importantly, Sdc-3 expression correlated with a macrophage gene signature in several cancer types. We found that TAMs infiltrating hypoxic tumors have significant expression of Sdc-3. Hypoxia directly induces Sdc-3 expression on macrophages. Interestingly, treatment with IFN-γ, but not IL-4, promotes Sdc-3 expression on macrophages. These findings are in line with the fact that IFN-γ stabilizes HIF-1α in macrophages (45).

Induction of Sdc-3 expression by IFN-γ indicates that Sdc-3 could be a marker of hot tumors. Hot tumors are characterized by a high degree of cytotoxic T cell infiltration that are the main source of IFN-γ. IFN-γ promotes tumor immune responses and limits cancer cell growth, but can also induce the expression of immune checkpoint molecules (i.e., PD-L1) or other immunosuppressive factors. In general, hot tumors have a better patient prognosis and predict the response to T cell checkpoint inhibition (48). The impact of tumor hypoxia in patient survival and response to therapies is less clear. Hypoxia inducible factor is known to promote a malignant tumor cell phenotype, metabolic alterations, and immunosuppressive pathways (i.e., adenosine pathway), but also to promote anti-tumor immune responses (33, 49).

Focusing on melanoma, a tumor that is considered immunogenic and in which expression of IFN-γ has a significant prognostic value, we show that expression of a hypoxia gene signature does not have an impact on patients OS. However, high expression of Sdc-3 on hypoxic tumors correlates with a better patient survival.

The impact of gene expression and function of hypoxia target genes in the TME is becoming a complex area of research with the potential of unraveling novel therapeutic targets. Little is known about the function of Sdc-3 in cancer progression and its influence on the immune response. Given the expression of Sdc-3 on endothelial cells and its role in remodeling the ECM, Sdc-3 might have an impact on immune cell infiltration, which could be modulated by therapeutic intervention. Moreover, the strong upregulation of Sdc-3 on tumor *versus* normal tissue indicates that this molecule could be exploited as a tumor-associated antigen in approaches based on cell therapy or antibody drug conjugates. Therefore, our findings support further investigation on the role of Sdc-3 in the TME to ascertain its potential use as a novel therapeutic target.

## Data Availability Statement

All datasets presented in this study are included in the article/Supplementary Material.

## Author Contributions

EP-F planned and executed experiments and wrote the manuscript. LE-M, SL, and AB executed experiments and performed flow cytometry analyses. AG, BJ-L, and AA-V performed data analyses. AP conceived and supervised the project and wrote the manuscript. All authors reviewed the manuscript.

## Conflict of Interest

The authors declare that the research was conducted in the absence of any commercial or financial relationships that could be construed as a potential conflict of interest.

## Footnotes

1 http://gepia2.cancer-pku.cn/

2 http://biogps.org/

## Abbreviations

ATCC: American Type Culture Collection
CAFs: cancer associated fibroblasts
CNS: central nervous system
CPM: counts per million
DLBC: diffuse large B-cell lymphoma
DMOG: dimethyloxalylglycine
ECM: extracellular matrix
gMFI: geometric mean fluorescence intensity
HIF: hypoxia inducible factor
HIF1-KO: HIF-1 α knockout CT26 murine cells
HRE: hypoxia response elements
IFN-γ: gamma interferon
IL-4: Interleukin-4
kDa: protein weight in kilodaltons
OS: overall survival
OV: ovarian serous cystadenocarcinoma
PAAD: pancreatic adenocarcinoma
PNS: peripheral nervous system
qPCR: quantitative PCR
scRNA-seq: single-cell RNA sequencing
Sdc: syndecan
Sdc-1: syndecan-1
Sdc-2: syndecan-2
Sdc-3: syndecan-3
Sdc-4: syndecan-4
sgRNA: single guide RNA
SKCM: skin cutaneous melanoma
TAMs: tumor associated macrophages
TCGA: The Cancer Genome Atlas program
TGCT: testicular germ cell tumors
THCA: thyroid carcinoma
THYM: thymoma
TME: tumor microenvironment
t-SNE: T-distributed stochastic neighbor embedding
WT: wild type.

## References

[1] Theocharis AD Skandalis SS Tzanakakis GN Karamanos NK. Proteoglycans in health and disease: novel roles for proteoglycans in malignancy and their pharmacological targeting. *FEBS J.* (2010) 277:3904–23. 10.1111/j.1742-4658.2010.07800.x 20840587

[2] Iozzo RV Karamanos N. Proteoglycans in health and disease: emerging concepts and future directions. *FEBS J.* (2010) 277:3863. 10.1111/j.1742-4658.2010.07796.x 20812984

[3] Iozzo RV Sanderson RD. Proteoglycans in cancer biology, tumour microenvironment and angiogenesis. *J Cell Mol Med.* (2011) 15:1013–31. 10.1111/j.1582-4934.2010.01236.x 21155971 PMC3633488

[4] Nikitovic D Berdiaki A Spyridaki I Krasanakis T Tsatsakis A Tzanakakis GN. Proteoglycans-biomarkers and targets in cancer therapy. *Front Endocrinol (Lausanne).* (2018) 9:69. 10.3389/fendo.2018.00069 29559954 PMC5845539

[5] Afratis NA Nikitovic D Multhaupt HA Theocharis AD Couchman JR Karamanos NK. Syndecans–key regulators of cell signaling and biological functions. *FEBS J.* (2017) 284:27–41. 10.1111/febs.13940 27790852

[6] Xian X Gopal S Couchman JR. Syndecans as receptors and organizers of the extracellular matrix. *Cell Tissue Res.* (2010) 339:31–46. 10.1007/s00441-009-0829-3 19597846

[7] Kirkpatrick CA Selleck SB. Heparan sulfate proteoglycans at a glance. *J Cell Sci.* (2007) 120:1829–32. 10.1242/jcs.03432 17515480

[8] Sarrazin S Lamanna WC Esko JD. Heparan sulfate proteoglycans. *Cold Spring Harb Perspect Biol.* (2011) 3:a004952. 10.1101/cshperspect.a004952 21690215 PMC3119907

[9] Bertrand J Bollmann M. Soluble syndecans: biomarkers for diseases and therapeutic options. *Br J Pharmacol.* (2019) 176:67–81. 10.1111/bph.14397 29931674 PMC6284332

[10] Couchman JR. Transmembrane signaling proteoglycans. *Annu Rev Cell Dev Biol.* (2010) 26:89–114. 10.1146/annurev-cellbio-100109-104126 20565253

[11] Vuong TT Reine TM Sudworth A Jenssen TG Kolset SO. Syndecan-4 is a major syndecan in primary human endothelial cells in vitro, modulated by inflammatory stimuli and involved in wound healing. *J Histochem Cytochem.* (2015) 63:280–92. 10.1369/0022155415568995 25575567 PMC4374057

[12] Yamada Y Arai T Kojima S Sugawara S Kato M Okato A Regulation of antitumor miR-144-5p targets oncogenes: direct regulation of syndecan-3 and its clinical significance. *Cancer Sci.* (2018) 109:2919–36. 10.1111/cas.13722 29968393 PMC6125479

[13] Kempf A Boda E Kwok JCF Fritz R Grande V Kaelin AM Control of cell shape, neurite outgrowth, and migration by a Nogo-A/HSPG interaction. *Dev Cell.* (2017) 43:24–34.e5. 10.1016/j.devcel.2017.08.014 28943240

[14] Arokiasamy S Balderstone MJM De Rossi G Whiteford JR. Syndecan-3 in inflammation and angiogenesis. *Front Immunol.* (2019) 10:3031. 10.3389/fimmu.2019.03031 31998313 PMC6962229

[15] de Witte L Bobardt M Chatterji U Degeest G David G Geijtenbeek TB Syndecan-3 is a dendritic cell-specific attachment receptor for HIV-1. *Proc Natl Acad Sci USA.* (2007) 104:19464–9. 10.1073/pnas.0703747104 18040049 PMC2148312

[16] De Rossi G Whiteford JR. A novel role for syndecan-3 in angiogenesis. *F1000Res.* (2013) 2:270. 10.12688/f1000research.2-270.v1 24555114 PMC3886797

[17] Cornelison DD Wilcox-Adelman SA Goetinck PF Rauvala H Rapraeger AC Olwin BB. Essential and separable roles for Syndecan-3 and Syndecan-4 in skeletal muscle development and regeneration. *Genes Dev.* (2004) 18:2231–6. 10.1101/gad.1214204 15371336 PMC517515

[18] Strader AD Reizes O Woods SC Benoit SC Seeley RJ. Mice lacking the syndecan-3 gene are resistant to diet-induced obesity. *J Clin Invest.* (2004) 114:1354–60. 10.1172/JCI20631 15520868 PMC524223

[19] Whitworth MK Backen AC Clamp AR Wilson G McVey R Friedl A Regulation of fibroblast growth factor-2 activity by human ovarian cancer tumor endothelium. *Clin Cancer Res.* (2005) 11:4282–8. 10.1158/1078-0432.CCR-04-1386 15958608

[20] Davies EJ Blackhall FH Shanks JH David G McGown AT Swindell R Distribution and clinical significance of heparan sulfate proteoglycans in ovarian cancer. *Clin Cancer Res.* (2004) 10:5178–86. 10.1158/1078-0432.CCR-03-0103 15297422

[21] Tinholt M Stavik B Louch W Carlson CR Sletten M Ruf W Syndecan-3 and TFPI colocalize on the surface of endothelial-, smooth muscle-, and cancer cells. *PLoS One.* (2015) 10:e0117404. 10.1371/journal.pone.0117404 25617766 PMC4305309

[22] Roskams T De Vos R David G Van Damme B Desmet V. Heparan sulphate proteoglycan expression in human primary liver tumours. *J Pathol.* (1998) 185:290–7. 10.1002/(SICI)1096-9896(199807)185:3<290::AID-PATH91>3.0.CO;2-I 9771483

[23] Marzioni D Lorenzi T Mazzucchelli R Capparuccia L Morroni M Fiorini R Expression of basic fibroblast growth factor, its receptors and syndecans in bladder cancer. *Int J Immunopathol Pharmacol.* (2009) 22:627–38. 10.1177/039463200902200308 19822079

[24] Bai PS Xia N Sun H Kong Y. Pleiotrophin, a target of miR-384, promotes proliferation, metastasis and lipogenesis in HBV-related hepatocellular carcinoma. *J Cell Mol Med.* (2017) 21:3023–43. 10.1111/jcmm.13213 28557334 PMC5661149

[25] Wu ZS Pandey V Wu WY Ye S Zhu T Lobie PE. Prognostic significance of the expression of GFRalpha1, GFRalpha3 and syndecan-3, proteins binding ARTEMIN, in mammary carcinoma. *BMC Cancer.* (2013) 13:34. 10.1186/1471-2407-13-34 23351331 PMC3562211

[26] Yao J Zhang LL Huang XM Li WY Gao SG. Pleiotrophin and N-syndecan promote perineural invasion and tumor progression in an orthotopic mouse model of pancreatic cancer. *World J Gastroenterol.* (2017) 23:3907–14. 10.3748/wjg.v23.i21.3907 28638231 PMC5467077

[27] Diamantopoulou Z Kitsou P Menashi S Courty J Katsoris P. Loss of receptor protein tyrosine phosphatase beta/zeta (RPTPbeta/zeta) promotes prostate cancer metastasis. *J Biol Chem.* (2012) 287:40339–49. 10.1074/jbc.M112.405852 23060448 PMC3504749

[28] Sun J Pan S Cui H Li H. CircRNA SCARB1 promotes renal cell carcinoma progression via miR-510-5p/SDC3 axis. *Curr Cancer Drug Targets.* (2020) 20:461–70. 10.2174/1568009620666200409130032 32271695

[29] Watanabe A Mabuchi T Satoh E Furuya K Zhang L Maeda S Expression of syndecans, a heparan sulfate proteoglycan, in malignant gliomas: participation of nuclear factor-kappaB in upregulation of syndecan-1 expression. *J Neuro Oncol.* (2006) 77:25–32. 10.1007/s11060-005-9010-3 16132527

[30] Labiano S Palazon A Melero I. Immune response regulation in the tumor microenvironment by hypoxia. *Semin Oncol.* (2015) 42:378–86. 10.1053/j.seminoncol.2015.02.009 25965356

[31] LaGory EL Giaccia AJ. The ever-expanding role of HIF in tumour and stromal biology. *Nat Cell Biol.* (2016) 18:356–65. 10.1038/ncb3330 27027486 PMC4898054

[32] Noman MZ Hasmim M Lequeux A Xiao M Duhem C Chouaib S Improving cancer immunotherapy by targeting the hypoxic tumor microenvironment: new opportunities and challenges. *Cells.* (2019) 8:1083. 10.3390/cells8091083 31540045 PMC6770817

[33] Palazon A Goldrath AW Nizet V Johnson RS. HIF transcription factors, inflammation, and immunity. *Immunity.* (2014) 41:518–28. 10.1016/j.immuni.2014.09.008 25367569 PMC4346319

[34] Gilkes DM Semenza GL Wirtz D. Hypoxia and the extracellular matrix: drivers of tumour metastasis. *Nat Rev Cancer.* (2014) 14:430–9. 10.1038/nrc3726 24827502 PMC4283800

[35] Lewis C Murdoch C. Macrophage responses to hypoxia: implications for tumor progression and anti-cancer therapies. *Am J Pathol.* (2005) 167:627–35. 10.1016/S0002-9440(10)62038-X 16127144 PMC1698733

[36] Henze AT Mazzone M. The impact of hypoxia on tumor-associated macrophages. *J Clin Invest.* (2016) 126:3672–9. 10.1172/JCI84427 27482883 PMC5096805

[37] Yang M McKay D Pollard JW Lewis CE. Diverse functions of macrophages in different tumor microenvironments. *Cancer Res.* (2018) 78:5492–503. 10.1158/0008-5472.CAN-18-1367 30206177 PMC6171744

[38] Melero I Berman DM Aznar MA Korman AJ Perez Gracia JL Haanen J. Evolving synergistic combinations of targeted immunotherapies to combat cancer. *Nat Rev Cancer.* (2015) 15:457–72. 10.1038/nrc3973 26205340

[39] Goldman MJ Craft B Hastie M Repecka K McDade F Kamath A Visualizing and interpreting cancer genomics data via the Xena platform. *Nat Biotechnol.* (2020) 38:675–8. 10.1038/s41587-020-0546-8 32444850 PMC7386072

[40] Iorio F Knijnenburg TA Vis DJ Bignell GR Menden MP Schubert M A landscape of pharmacogenomic interactions in cancer. *Cell.* (2016) 166:740–54. 27397505 10.1016/j.cell.2016.06.017 PMC4967469

[41] Su AI Wiltshire T Batalov S Lapp H Ching KA Block D A gene atlas of the mouse and human protein-encoding transcriptomes. *Proc Natl Acad Sci USA.* (2004) 101:6062–7. 10.1073/pnas.0400782101 15075390 PMC395923

[42] Mabbott NA Baillie JK Brown H Freeman TC Hume DA. An expression atlas of human primary cells: inference of gene function from coexpression networks. *BMC Genomics.* (2013) 14:632. 10.1186/1471-2164-14-632 24053356 PMC3849585

[43] Ye Y Hu Q Chen H Liang K Yuan Y Xiang Y Characterization of hypoxia-associated molecular features to aid hypoxia-targeted therapy. *Nat Metab.* (2019) 1:431–44. 10.1038/s42255-019-0045-8 31984309 PMC6980239

[44] Tirosh I Izar B Prakadan SM Wadsworth MH II Treacy D Trombetta JJ Dissecting the multicellular ecosystem of metastatic melanoma by single-cell RNA-seq. *Science.* (2016) 352:189–96. 27124452 10.1126/science.aad0501 PMC4944528

[45] Takeda N O’Dea EL Doedens A Kim JW Weidemann A Stockmann C Differential activation and antagonistic function of HIF-{alpha} isoforms in macrophages are essential for NO homeostasis. *Genes Dev.* (2010) 24:491–501. 10.1101/gad.1881410 20194441 PMC2827844

[46] Fujita N Hirose Y Tran CM Chiba K Miyamoto T Toyama Y HIF-1-PHD2 axis controls expression of syndecan 4 in nucleus pulposus cells. *FASEB J.* (2014) 28:2455–65. 10.1096/fj.13-243741 24558194 PMC4021441

[47] Eustace AD McNaughton EF King S Kehoe O Kungl A Mattey D Soluble syndecan-3 binds chemokines, reduces leukocyte migration in vitro and ameliorates disease severity in models of rheumatoid arthritis. *Arthritis Res Ther.* (2019) 21:172. 10.1186/s13075-019-1939-2 31300004 PMC6625118

[48] Galon J Bruni D. Approaches to treat immune hot, altered and cold tumours with combination immunotherapies. *Nat Rev Drug Discov.* (2019) 18:197–218. 10.1038/s41573-018-0007-y 30610226

[49] Petrova V Annicchiarico-Petruzzelli M Melino G Amelio I. The hypoxic tumour microenvironment. *Oncogenesis.* (2018) 7:10. 10.1038/s41389-017-0011-9 29362402 PMC5833859

