# Supplementary material for: Hypoxia Promotes Syndecan-3 Expression in the Tumor Microenvironment
Source: Front Immunol. 2020 Sep 30;11:586977. doi: 10.3389/fimmu.2020.586977 (PMC7561406; doi:10.3389/fimmu.2020.586977)
Supplement: Supplementary file 1 [file Data_Sheet_1.DOCX]

Supplementary Material


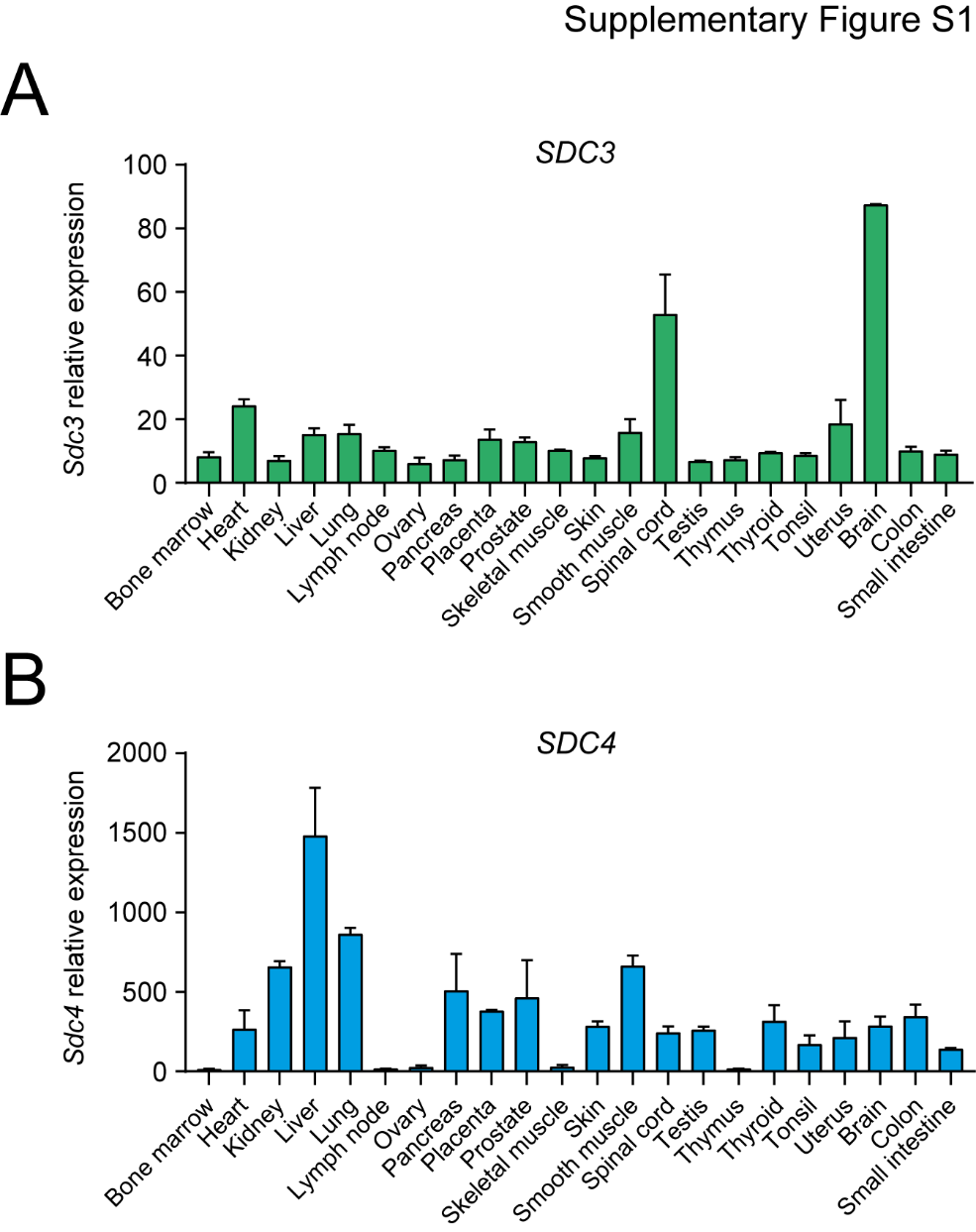


**Supplementary Figure 1**. The expression of Sdc-3 on healthy tissue is confined to spinal cord and brain while the expression of Sdc-4 is broader. **(A,B)** Relative expression of *SDC3* (A) and *SDC4* (B) genes across different healthy tissues. Data (mean and SD) were extracted from the GeneAtlas U133A dataset through the BioGPS portal.


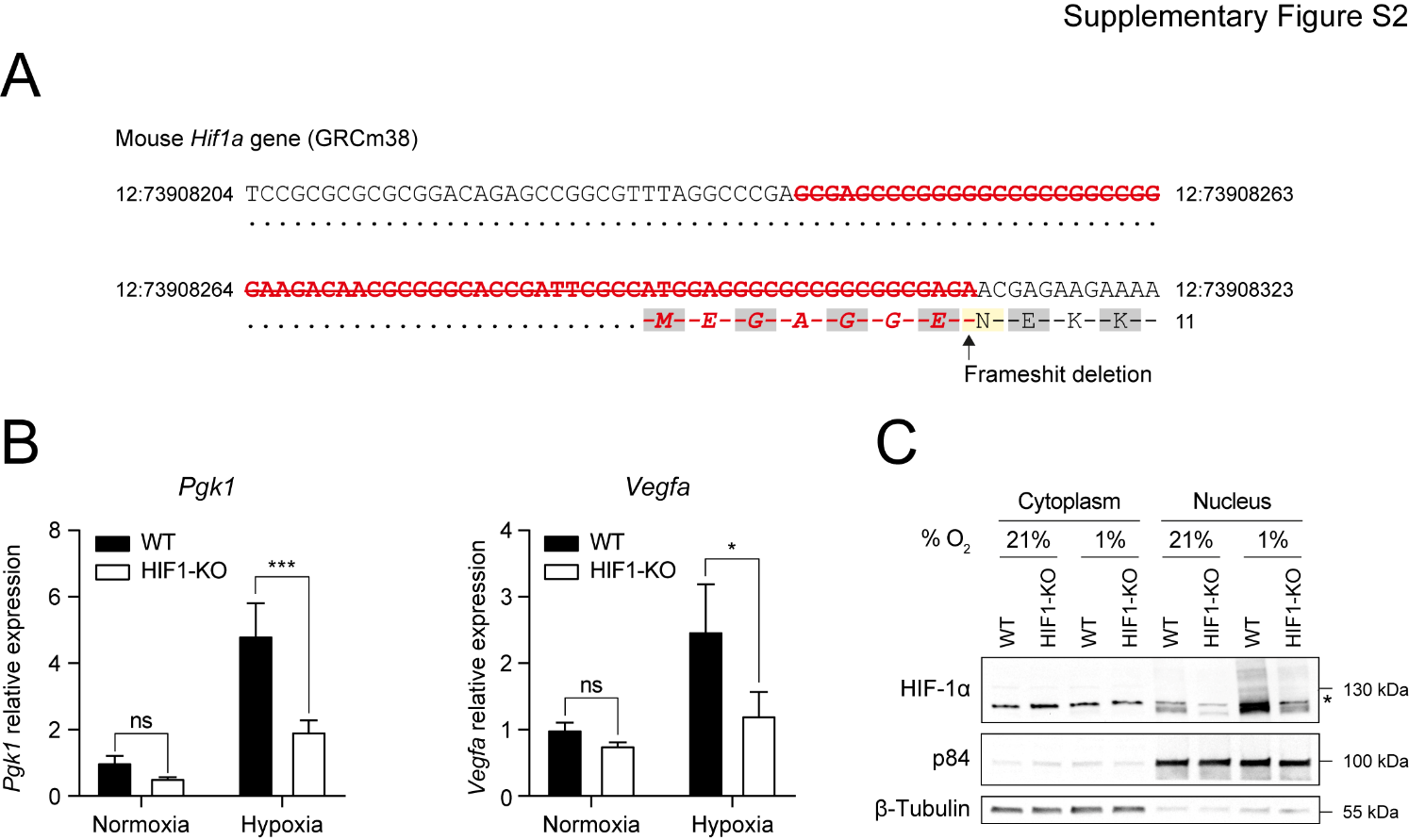


**Supplementary Figure 2**. Characterization of the HIF1-KO CT26 tumor cell line. **(A)** The presented DNA sequence corresponds to the CRISPR-edited region of mouse *Hif1a* gene. Sanger sequencing confirmed a 72 bp deletion, shown in red, that included the sequence encoding the first seven aminoacids of exon 1. The next codon, which codifies for the asparagine, shown in yellow, was affected by a frameshift deletion. **(B)** Expression of mouse *Pgk1* (left panel) and *Vegfa* (right panel) genes on WT and HIF1-KO CT26 cells cultured under normoxia (21% oxygen) or hypoxia (1% oxygen) for 24 hours. One representative experiment is shown; error bars represent SD (two-way ANOVA test). Asterisks represent p values for each statistical test as follows: ns (P>0.05), * (P ≤ 0.05) and *** (P ≤ 0.001). **(C)** Western blot analysis of cytoplasmic and nuclear fractions extracted from WT and HIF1-KO CT26 cells cultured under normoxia or hypoxia for 4 hours and probed with antibodies against HIF-1α, nuclear matrix protein p84 and β-tubulin. The asterisk represents a non-specific band.

**Supplementary Table 1**. Number (N) for each of the 21 tumor types and normal or adjacent tissue samples included in this study. Source: TCGA and GTEx datasets.

| **TCGA abbreviation** | **Cancer type** | **N tumor** | **N control** |
| --- | --- | --- | --- |
| BLCA | Bladder urothelial carcinoma | 407 | 28 |
| DLBC | Lymphoid neoplasm Diffuse Large B-Cell lymphoma | 47 | 337 |
| KICH | Kidney chromophobe | 66 | 57 |
| LAML | Acute myeloid leukemia | 173 | 70 |
| THCA | Thyroid carcinoma | 512 | 338 |
| CESC | Cervical squamous cell carcinoma and endocervical adenocarcinoma | 306 | 13 |
| ESCA | Esophageal carcinoma | 182 | 258 |
| GBM | Glioblastoma multiforme | 166 | 208 |
| HNSC | Head and neck squamous cell carcinoma | 520 | 44 |
| KIRC | Kidney renal clear cell carcinoma | 530 | 100 |
| LGG | Brain lower grade glioma | 523 | 208 |
| OV | Ovarian serous cystadenocarcinoma | 427 | 88 |
| PAAD | Pancreatic adenocarcinoma | 179 | 171 |
| READ | Rectum adenocarcinoma | 93 | 10 |
| SKCM | Skin cutaneous melanoma | 469 | 557 |
| STAD | Stomach adenocarcinoma | 414 | 211 |
| TGCT | Testicular germ cell tumors | 154 | 165 |
| THYM | Thymoma | 119 | 338 |
| UCEC | Uterine corpus endometrial carcinoma | 181 | 93 |
| COAD | Colon adenocarcinoma | 290 | 349 |
| LUAD | Lung adenocarcinoma | 515 | 347 |

**Supplementary Table 2**. Primer sequences used for mRNA quantification by qPCR. Abbreviations: Forward primer (F); Reverse primer (R).

| **Organism** | **Gene** | **Sequence** (5' to 3') | | **Amplicon size** (bp) | **Source** (PubMed ID) |
| --- | --- | --- | --- | --- | --- |
| Mouse | *Arg1* | F: | AACACGGCAGTGGCTTTAACC | 117 | 22158945 |
|  |  | R: | GGTTTTCATGTGGCGCATTC |  |  |
|  | *Epas1* | F: | CAACCTGCAGCCTCAGTGT | 144 | This paper |
|  |  | R: | CACCACGTCGTTCTTCTCGA |  |  |
|  | *Hif1a* | F: | GAAACGACCACTGCTAAGGCA | 129 | This paper |
|  |  | R: | GGCAGACAGGTTAAGGCTCCT |  |  |
|  | *Hif1a-KO* | F: | CTGGACTTGTCTCTTTCTCCGC | 100 | This paper |
|  |  | R: | GAATCGGTGCCCGCGTT |  |  |
|  | *Nos2 (iNOS)* | F: | CGAAACGCTTCACTTCCAA | 51 | 22158945 |
|  |  | R: | TGAGCCTATATTGCTGTGGCT |  |  |
|  | *Pgk1* | F: | ATTCTGCTTGGACAATGGAGC | 76 | This paper |
|  |  | R: | AGGCATGGGAACACCATCA |  |  |
|  | *Rplp0* | F: | CGACCTGGAAGTCCAACTAC | 109 | This paper |
|  |  | R: | ATCTGCTGCATCTGCTTG |  |  |
|  | *Sdc1* | F: | TCTGGGGATGACTCTGACAAC | 68 | 18342939 |
|  |  | R: | TGCCGTGACAAAGTATCTGG |  |  |
|  | *Sdc2* | F: | TTCAGGAGTATATCCTATTGATGATGA | 76 | 18342939 |
|  |  | R: | ACTCTCTATGTCTTCATCAGCTCCT |  |  |
|  | *Sdc3* | F: | GAGGAGTACCCTGCCGTTG | 71 | 18342939 |
|  |  | R: | ACTCTGGAGTTGGGGTCTGA |  |  |
|  | *Sdc4* | F: | CCCTTCCCTGAAGTGATTGA | 98 | 18342939 |
|  |  | R: | AGTTCCTTGGGCTCTGAGG |  |  |
|  | *Serpine1* (*PAI1*) | F: | CCTTGCTTGCCTCATCCTGG | 406 | 27258009 |
|  |  | R: | CTGGAAGAGCTTGAAGAAGTGG |  |  |
|  | *Slc2a1* (*Glut1*) | F: | CATCCTTATTGCCCAGGTGTTT | 82 | 27918549 |
|  |  | R: | GAAGACGACACTGAGCAGCAGA |  |  |
|  | *Vegfa* | F: | CCACGTCAGAGAGCAACATCA | 75 | This paper |
|  |  | R: | TCATCTCTCCTATGTGCTGGCTTT |  |  |

**Supplementary Table 3**. mRNA expression correlation analyses of *SDC3* and other genes. Spearman and Pearson correlation coefficients r and their corresponding P values are shown. Significant (p<0.05) negative (red) or positive (blue) correlations are highlighted. Pearson correlation coefficient r ≥ 0.3 or ≤ -0.3 and Spearman correlation coefficient r ≥ 0.4 or ≤ -0.4 are shown in bold. Abbreviations: Pearson (Pe); Correlation coefficient (r); Spearman (Sp).

**Supplementary Table 4**. mRNA expression correlation analyses of *SDC4* and other genes. Spearman and Pearson correlation coefficients r and their corresponding P values are shown. Significant (p<0.05) negative (red) or positive (blue) correlations are highlighted. Pearson correlation coefficient r ≥ 0.3 or ≤ -0.3 and Spearman correlation coefficient r ≥ 0.4 or ≤ -0.4 are shown in bold. Abbreviations: Pearson (Pe); Correlation coefficient (r); Spearman (Sp).
